# Methyl jasmonate effects on sugarbeet root responses to postharvest dehydration

**DOI:** 10.7717/peerj.11623

**Published:** 2021-06-17

**Authors:** Fernando L. Finger, John D. Eide, Abbas M. Lafta, Mohamed F.R. Khan, Munevver Dogramaci, Karen K. Fugate

**Affiliations:** 1 Departamento de Agronomia, Universidade Federal de Viçosa, Viçosa, MG, Brazil; 2 Edward T. Schafer Agricultural Research Center, USDA-ARS, Fargo, ND, United States of America; 3 Department of Plant Pathology, North Dakota State University, Fargo, ND, United States of America; 4 Extension Service, University of Minnesota, St. Paul, MN, United States of America

**Keywords:** *Beta vulgaris* L., Proline, Storage, Respiration, Sprouting, Water stress, Wound healing

## Abstract

**Background:**

Sugarbeet (*Beta vulgaris* L.) roots are stored under conditions that cause roots to dehydrate, which increases postharvest losses. Although exogenous jasmonate applications can reduce drought stress in intact plants, their ability to alleviate the effects of dehydration in postharvest sugarbeet roots or other stored plant products is unknown. Research was conducted to determine whether jasmonate treatment could mitigate physiological responses to dehydration in postharvest sugarbeet roots.

**Methods:**

Freshly harvested sugarbeet roots were treated with 10 µM methyl jasmonate (MeJA) or water and stored under dehydrating and non-dehydrating storage conditions. Changes in fresh weight, respiration rate, wound healing, leaf regrowth, and proline metabolism of treated roots were investigated throughout eight weeks in storage.

**Results:**

Dehydrating storage conditions increased root weight loss, respiration rate, and proline accumulation and prevented leaf regrowth from the root crown. Under dehydrating conditions, MeJA treatment reduced root respiration rate, but only in severely dehydrated roots. MeJA treatment also hastened wound-healing, but only in the late stages of barrier formation. MeJA treatment did not impact root weight loss or proline accumulation under dehydrating conditions or leaf regrowth under non-dehydrating conditions. Both dehydration and MeJA treatment affected expression of genes involved in proline metabolism. In dehydrated roots, proline dehydrogenase expression declined 340-fold, suggesting that dehydration-induced proline accumulation was governed by reducing proline degradation. MeJA treatment altered proline biosynthetic and catabolic gene expression, with greatest effect in non-dehydrated roots. Overall, MeJA treatment alleviated physiological manifestations of dehydration stress in stored roots, although the beneficial effects were small. Postharvest jasmonate applications, therefore, are unlikely to significantly reduce dehydration-related storage losses in sugarbeet roots.

## Introduction

Dehydration during storage reduces quality for nearly all harvested plant products. Dehydration begins when plant products are separated from their preharvest sources of hydration and continues throughout storage unless high humidity conditions are maintained. Physical changes associated with water loss include reductions in weight, turgidity, and firmness which negatively impact product acceptability and economic return (Paull, 1999; Bryant, 2012; Holcroft, 2015). Metabolism is also altered by water stress, which causes alterations in gene expression, protein profiles, endogenous hormone levels, and concentrations of carbohydrates, osmotically active compounds, secondary metabolites, and cell wall components (Somboonkaew & Terry, 2010; Bonghi et al., 2012; Romero et al., 2012). Like most plant products, sugarbeet (*Beta vulgaris* L.) roots are negatively impacted by postharvest water loss. For this crop, dehydration elevates root sucrose loss, respiration rate, storage rot losses, softening, and the formation of invert sugars which hinder sugarbeet root processing (Vukov & Hangyál, 1985; Tungland, Watkins & Schmidt, 1998; Lafta & Fugate, 2009).

Although controlled humidity storage can minimize or prevent postharvest water loss, such storage conditions are not economically feasible for sugarbeet roots. Instead, sugarbeet roots are stored in large outdoor piles or sheds that are cooled using the cold ambient air of late autumn and winter (Campbell & Klotz, 2006; Bernhardson, 2009). While the passage of cold, dry winter air through storage piles provides an economical way to cool millions of tons of sugarbeet roots, it inevitably dehydrates roots. Roots located at the surface of piles are also exposed to sun, wind, and freeze/thaw cycles. These roots suffer extreme dehydration and lose 40% or more of their weight during storage (Tungland, Watkins & Schmidt, 1998). Although sugarbeet storage piles are sometimes covered to alleviate environmental stress, these coverings reduce, but do not eliminate, root dehydration (Tungland, Watkins & Schmidt, 1998; Campbell & Klotz, 2006).

Jasmonic acid (JA) and JA derivatives including methyl jasmonate (MeJA) are endogenous hormones that are synthesized in response to drought and mediate plant drought-stress responses (Seo et al., 2011; Andrade et al., 2017). Applied exogenously, these compounds alleviate the negative effects of drought stress in many plant species by altering water uptake, promoting water conservation, and protecting plants from reactive oxygen species (Sánchez-Romera et al., 2014; Riemann et al., 2015; Anjum et al., 2016). In sugarbeet, MeJA treatment alleviates drought-stress in young plants by allowing them to maintain higher water content, delaying the onset of drought stress, and mitigating the physiological effects of stress (Fugate et al., 2018). MeJA application also affects the accumulation of proline, an osmotically active compound that protects against drought stress by promoting cellular hydration, stabilizing membranes, and scavenging reactive oxygen species (Ashraf & Foolad, 2007; Hayat et al., 2012; Fugate et al., 2018).

Beneficial effects of jasmonate treatments are documented for many harvested plant products (Tripathi & Dubey, 2004; Rohwer & Erwin, 2008). Postharvest MeJA treatments reduce chilling injury in cold-sensitive plant products, decrease storage disease in many products, promote wound healing in potato tubers, reduce shoot regrowth in radish roots, and delay ripening and senescence in eggplant fruits (Wang, 1998; Tripathi & Dubey, 2004; Ozeretskovskaya et al., 2009; Zhang et al., 2012; Guo et al., 2014; Fan et al., 2016). In sugarbeet, postharvest jasmonate treatments protect roots from storage rot pathogens (Fugate et al., 2012). The ability of jasmonate to alleviate other forms of postharvest stress in sugarbeet roots, however, has not been examined.

Because of MeJA’s proven ability to alleviate drought stress in young sugarbeet plants and reduce postharvest losses from a variety of abiotic, biotic, and physiological causes, research was conducted to determine whether a postharvest MeJA treatment could mitigate dehydration effects on stored sugarbeet roots. For these experiments, harvested roots were treated with MeJA at a concentration previously determined to be optimal for jasmonate responses in stored sugarbeet roots (Fugate et al., 2012). Roots were then stored under dehydrating and non-dehydrating storage conditions and storage traits, including weight loss, respiration rate, wound-healing rate, and leaf regrowth were determined during eight weeks in storage. Additionally, proline accumulation and the transcription of genes involved in proline synthesis and degradation were determined since proline concentrations are typically altered by dehydration and can be affected by MeJA treatment. Overall, the purpose of this research was to determine the feasibility of using MeJA to reduce sugarbeet storage losses by evaluating its effects on dehydration-induced changes in root weight, respiration rate, leaf regrowth, and wound healing.

### Materials and Methods

#### Plant material and treatments

A total of 128 sugarbeet (*Beta vulgaris* L.) plants of variety VDH66156 (SESVanderHave, Tienen, Belgium) were grown in a greenhouse in 15 L pots under 16 h day and 8 h night periods. After 16 weeks, taproots were harvested, and shoots were removed with a knife. Shoot removal left no petiole material attached to the root but caused a small, flat, transverse wound on the taproot apex. Roots were gently washed to remove adhering soil that might promote disease, increase root weight, and contaminate samples collected for RNA analysis. Washed roots were allowed to dry at room temperature for 2 h and randomly assigned to two groups. One group was immersed in 10 µM MeJA (Cayman Chemical Co., Ann Arbor, MI, USA) for 1 h at room temperature (RT). The second group was immersed in water for 1 h at RT as controls. After treatments, roots were placed into perforated polyethylene plastic bags with eight similarly treated roots per bag. Half of the bags for each treatment (MeJA or control) were stored in a growth chamber (Conviron, model PGR 15, Winnipeg, MB, Canada) operating at 20 °C and 78% relative humidity as a low humidity storage treatment; the remaining bags were stored in a similar growth chamber operating at 20 °C and 98% relative humidity as a high humidity storage treatment. Roots were stored at 20 °C to mimic suboptimal storage conditions that commonly occur in commercial piles and which accelerate storage deterioration. Roots of both treatments were stored for up to eight weeks. Relative humidity inside perforated bags was monitored at 2 h intervals using HOBO data loggers (Onset Computer Corp, Bourne, MA, USA). The average relative humidity inside polyethylene bags that were incubated in the low relative humidity chamber was 90.5%. Average relative humidity in bags incubated in the high relative humidity treatment was 98.0%. Individual roots were the experimental unit for all analyses. Eight replicates were used for all analyses except quantitative real-time PCR (qRT-PCR) analyses which were conducted with three replicates. For chemical and molecular analyses, root samples were collected after 0, 4, 6, and 8 weeks in storage. Tissue was collected from roots where root girth was widest with care taken to avoid periderm tissue. Collected tissue was flash frozen in liquid nitrogen, lyophilized, and stored at −80 °C prior to use.

#### Respiration rate and vapor conductance

Respiration rate and vapor conductance of individual roots were determined after 0, 1, 2, 3, 4, 6, and 8 weeks in storage, using the same roots for all sampling times. To determine root respiration rate, roots were weighed, and the CO_2_ produced by the root was determined by infrared gas analysis using an open system with a continuous, 1,000 µmol s^−1^ flow of air. The apparatus used for respiration determinations comprised a 7 L sample chamber attached to the air pump and gas analyzer from a LI-COR 6400XT photosynthesis system (Lincoln, NE, USA) as previously described by Haagenson et al. (2006). Vapor conductance was measured at the wounded surface of the taproot apex using a LI-COR model LI-1600M steady-state porometer.

#### Proline determinations

Proline concentration was determined as described by *Bates, Waldren & Teare (1973)*. Lyophilized tissue (30 mg) was mixed with 1.0 mL of 3% sulfosalicylic acid, sonicated for 15 min at RT and centrifuged for 30 min at 16,000 g. The resulting supernatant was reacted with 3% glacial acetic acid and acid ninhydrin at 95 °C for 1 h, cooled to RT, and extracted with 0.8 mL of toluene. After vortexing for 15 s, the toluene layer was removed and the absorbance at 520 nm was measured against a standard curve generated with L-proline solutions of known concentration.

#### RNA extraction and qRT-PCR analyses

Total RNA was extracted from 50 mg of lyophilized tissue using a RNeasy Plant Mini Kit (QIAGEN, Valencia, CA, USA) with an on-column DNase digestion. cDNA was produced from 100 ng RNA using Maxima H Minus First Strand cDNA Synthesis Kit with dsDNase (ThermoFisher Scientific, Waltham, MA, USA). Expression level changes for the genes of proline metabolism, Δ^1^–pyrroline-5-carboxylate synthetase (P5CS), Δ^1^–pyrroline-5-carboxylate reductase (P5CR), ornithine aminotransferase (OAT), and proline dehydrogenase (PDH) were determined by qRT-PCR relative to the expression of two housekeeping genes, ubiquitin (UBQ) and glyceraldehyde-3-phosphate dehydrogenase (G3PDH) using the primer pairs in Table 1. Primer pairs for P5CS, P5CR, OAT, and PDH were designed with Primer3Plus (Untergasser et al., 2007); primers for UBQ and G3PDH were obtained from Hébrard et al. (2015) and Liebe & Varrelmann (2018), respectively. Efficiency of all primers were determined prior to use. A MJ Research PTC-200 thermal cycler (Watertown, MA, USA) equipped with a Bio-Rad Laboratories Chromo 4 detector (Hercules, CA, USA) was used to perform qRT-PCR reactions using Power SYBR Green PCR Master Mix (Applied Biosystems, Foster City, CA, USA), 100 ng cDNA and 250 nM forward and reverse primers, and a program that denatured samples for 10 min at 95 °C and amplified products using 40 cycles of 15 s at 95 °C and 60 s at 60 °C. Three replicate reactions were performed for each gene with the average Ct for the three reactions used to calculate expression. Changes in expression were calculated using the methods of *Pfaffl (2001)*.

**Table 1:** Genes and primer sequences used in qRT-PCR reactions.

Gene	GenBank identifier	Encoded protein	Primers (5′→ 3′)
*BvP5CS*	LOC104904817	Δ^1^–pyrroline-5-carboxylate synthetase	F: TGCCGTTGTTACAAGGAGTG R: CCTAAGCCTCTGACGACCAG
*BvP5CR*	LOC104889452	Δ^1^–pyrroline-5-carboxylate reductase	F: GAGGGACAGCAACTGAGGAG R: ACCTTTGGCAAGTTCTCGTG
*BvOAT*	LOC104901265	ornithine aminotransferase	F: GGCGAGGAGAAGATTATTGC R: CCCTTGCTTGAGAGAACACC
*BvPDH*	LOC104905171	proline dehydrogenase	F: GCTGGTTTTCAAGTGAGCAAG R: AACTCCATCCCCATGAGTTG
*BvUBQ*	LOC104907074	ubiquitin	F: TCGAAGATGGCCGTACTTTGGC R: CCCTCAAACGGAGAACCAAGTG
*BvG3PDH*	LOC104893518	glyceraldehyde-3-phosphate dehydrogenase	F: CACCACCGATTACATGACATACA R: GGATCTCCTCTGGGTTCCTG

#### Statistical analysis

Significant differences between treatments were determined by analysis of variance (ANOVA) using Minitab software (ver. 19, State College, PA, USA). Tukey’s multiple comparison test was used at significance level *α* = 0.05 to compare differences between means. Regression analyses were conducted using statistical functions within Excel 365 (Microsoft, Redmond, WA, USA) with best-fit trendlines chosen to maximize the coefficient of determination.

## Results

### Weight loss

**Figure 1:**
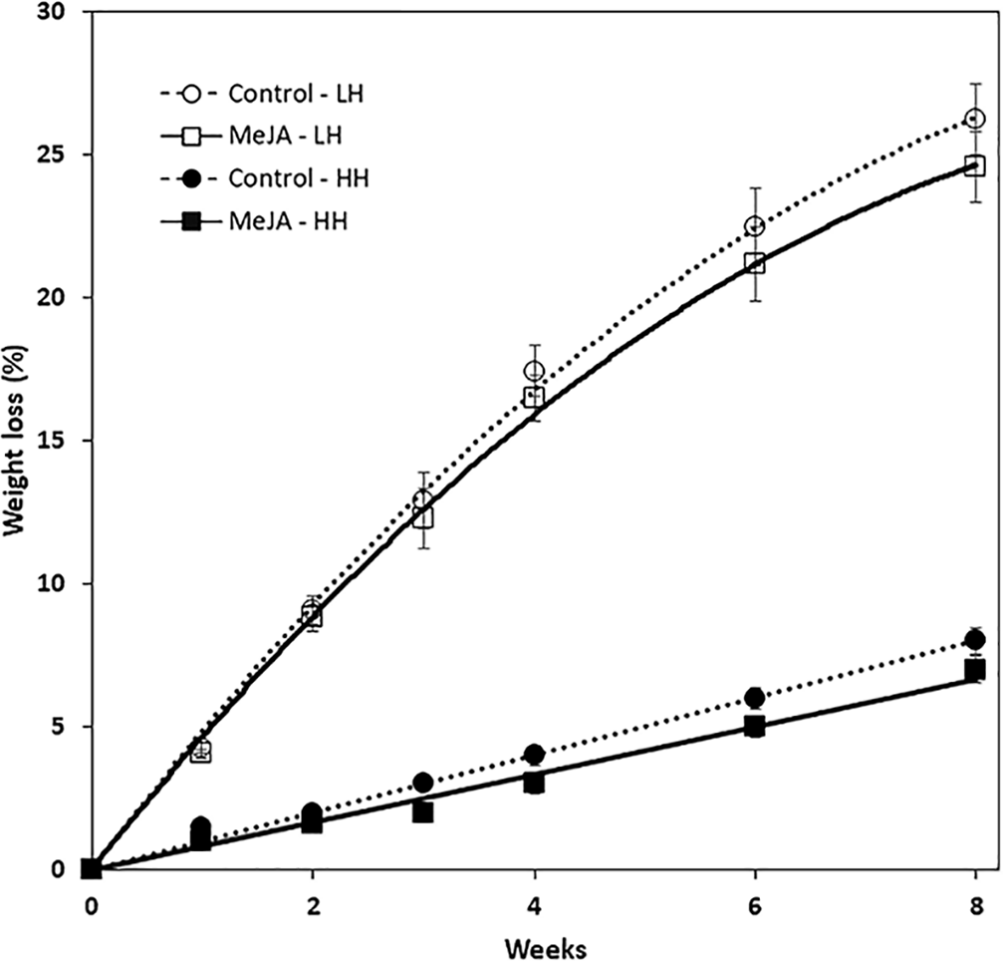
Accumulated weight loss of methyl jasmonate (MeJA)-treated and water-treated (control) sugarbeet roots stored under high humidity (HH) and low humidity (LH) conditions. Roots were treated with 10 µM MeJA or water after harvest and stored at 20 °C. High and low humidity conditions were 98% and 91%, respectively. Data points are means ± SE of the means, with eight replicates. Where error bars are not apparent, SE was smaller than the symbol for the data point. Equations for regression lines are available in File S1.

MeJA, applied as a 10 µM solution to harvested roots, reduced weight loss during storage (Fig. 1). The reduction in weight loss from MeJA, however, was minimal and statistically significant only for roots that were stored under high humidity conditions. Under these conditions, MeJA-treated roots lost 1% less of their weight after eight weeks storage relative to controls. In contrast, relative humidity conditions strongly influenced weight loss during storage and after eight weeks, roots stored at low and high relative humidity levels of 91 and 98% lost 25 and 5%, respectively, of their fresh weight. Under low humidity conditions, weight loss was sufficiently severe that roots lost turgidity and were flexible.

### Root respiration rate

**Figure 2:**
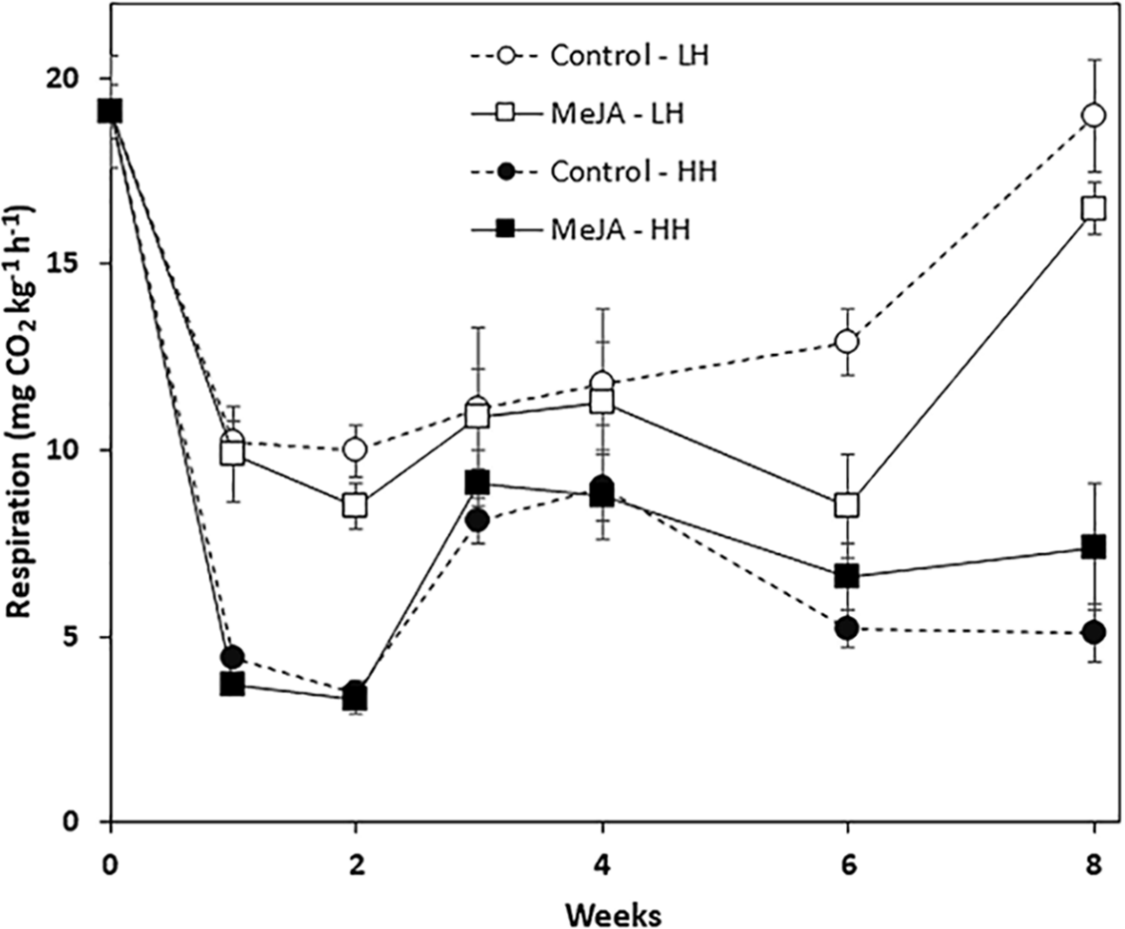
Respiration rate of methyl jasmonate (MeJA)-treated and water-treated (control) sugarbeet roots stored under high humidity (HH) and low humidity (LH) conditions. Roots were treated with 10 µM MeJA or water after harvest and stored at 20 °C. High and low humidity conditions were 98% and 91%, respectively. Data points are means ± SE of the means, with eight replicates. Where error bars are not apparent, SE was smaller than the symbol for the data point.

Storage duration, relative humidity conditions, and a pre-storage MeJA treatment all affected root respiration rate, although MeJA effects were evident only in roots stored for prolonged periods at low humidity (Fig. 2). Regardless of treatment, respiration rates declined sharply during the first week in storage and remained stable during the following week. For roots stored at low humidity, respiration rate declined during the first week by 48%, while roots stored at high humidity declined by 81% during this same period. For all time points, roots at high humidity respired at lower rates than roots stored under low humidity conditions. MeJA treatment had no significant effect on root respiration under high humidity storage conditions. Under low humidity conditions, MeJA treatment reduced respiration rate, but only after roots had been stored for 6 and 8 weeks. At these time points, MeJA treatment reduced root respiration by 34 and 13%, respectively, relative to controls.

### Wound healing

**Figure 3:**
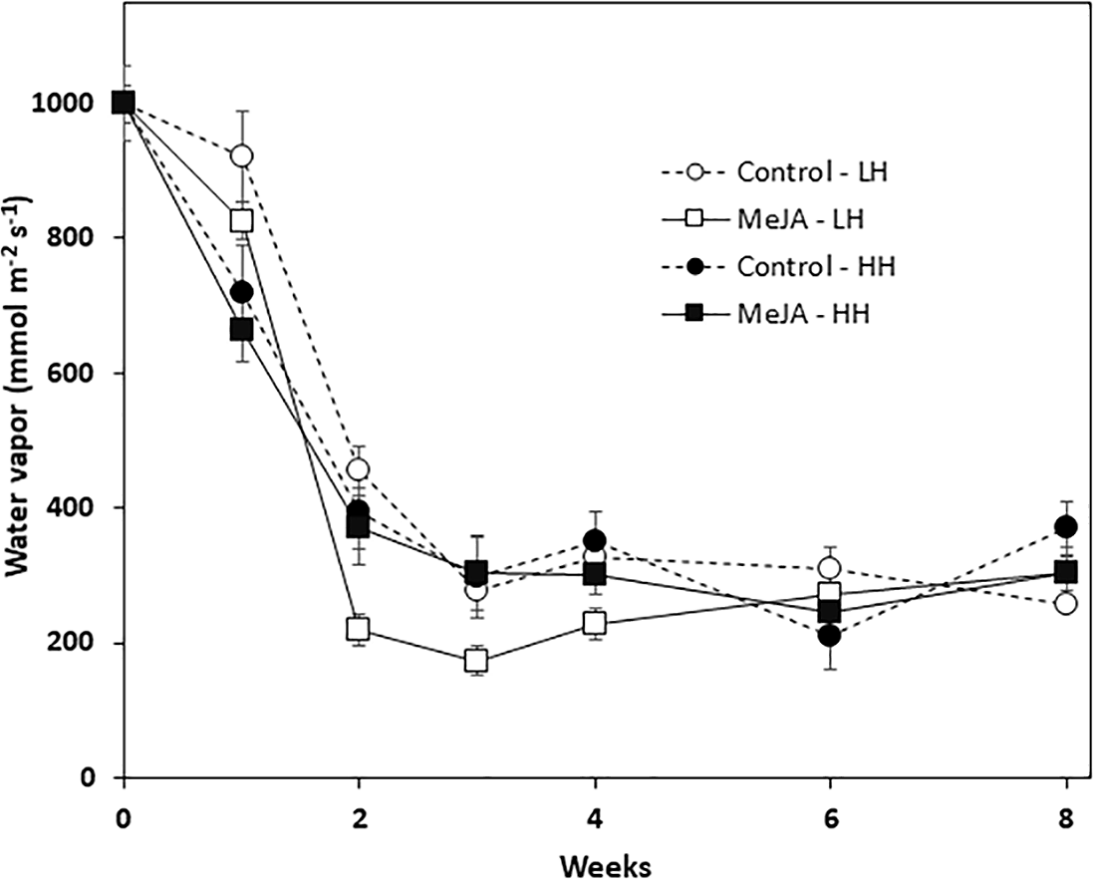
Vapor conductance from the wounded apex of sugarbeet roots treated with methyl jasmonate (MeJA) or water (control) at harvest and during storage under high humidity (HH) and low humidity (LH) conditions. Roots were treated with 10 µM MeJA or water after harvest and stored at 20 °C. High and low humidity conditions were 98% and 91%, respectively. Data points are means ± SE of the means, with eight replicates.

Wound healing was quantified by determining the vapor conductance at the wound site that was created by shoot removal at harvest. Vapor conductance declined sharply during the first two weeks in storage for all roots, regardless of MeJA treatment or storage humidity conditions (Fig. 3). The decline in vapor conductance, however, was greatest for MeJA-treated roots stored at low relative humidity. In these roots, vapor conductance rates after 2, 3, and 4 weeks storage were significantly lower than those of roots from all other treatments. Nevertheless, after 6 and 8 weeks in storage, vapor conductance and water loss from the wounded surface were similar for all roots regardless of MeJA treatment or the humidity of storage conditions.

### Leaf regrowth

Although leaf material was completely removed at harvest, some of the vegetative buds on the crown of the root remained and these regrew into shoots on roots stored at high relative humidity. Shoot regrowth, however, did not occur in roots stored under low relative humidity conditions. For high-humidity roots stored for eight weeks, 12.5% of the roots that received a MeJA treatment had visible sprouts with an average leaf length of 19.8 ± 5.9 mm. For the high-humidity controls, 16.1% of roots had sprouts with a mean leaf length of 22.5 ± 7.9 mm. Although sprouting incidence and leaf length were slightly greater in controls relative to MeJA-treated roots, MeJA treatment had no statistically significant effect on sprouting.

### Proline metabolism

#### Accumulation

Proline concentration increased during storage for all roots except for the high-humidity, water-treated controls (Table 2). At all timepoints during storage, proline concentrations were higher in low-humidity roots than in high-humidity stored roots. Under low-humidity conditions, proline concentrations increased significantly after 4 weeks and were elevated by 2.2 and 2.0-fold in MeJA-treated and control roots, respectively, after eight weeks in storage. In contrast, proline concentration after eight weeks under high-humidity conditions, increased by only 1.5-fold in MeJA-treated roots and was not significantly altered in control roots. Proline concentrations were generally greater in MeJA-treated roots than controls, regardless of humidity conditions. However, differences due to MeJA treatment were not statistically significant.

**Table 2:** Proline concentration of sugarbeet roots treated with methyl jasmonate (MeJA) or water (control) during storage under high and low humidity conditions^*^.

Weeks in storage	Low humidity		High humidity	
	Control	MeJA	Control	MeJA
0	0.91 Ac^**^	0.91 Ac	0.91 Aa	0.91 Ab
4	1.53 Ab	1.56 Ab	1.04 Ba	1.01 Bab
6	1.76 Aab	1.57 Ab	0.99 Ba	1.11 Bab
8	1.83 Aa	2.03 Aa	1.07 Ba	1.32 Ba

**Notes.**

* Proline concentration expressed as mg proline per g dry weight. Roots were treated with 10 μM MeJA or water as a control on the day of harvest and stored under low humidity (91%) or high humidity (98%) conditions at 20 °C for up to 8 weeks.

** Means followed by the same uppercase letter within rows and by the same lowercase letter within columns do not differ by Tukey’s test at 5% probability.

#### qRT-PCR analyses

**Figure 4:**
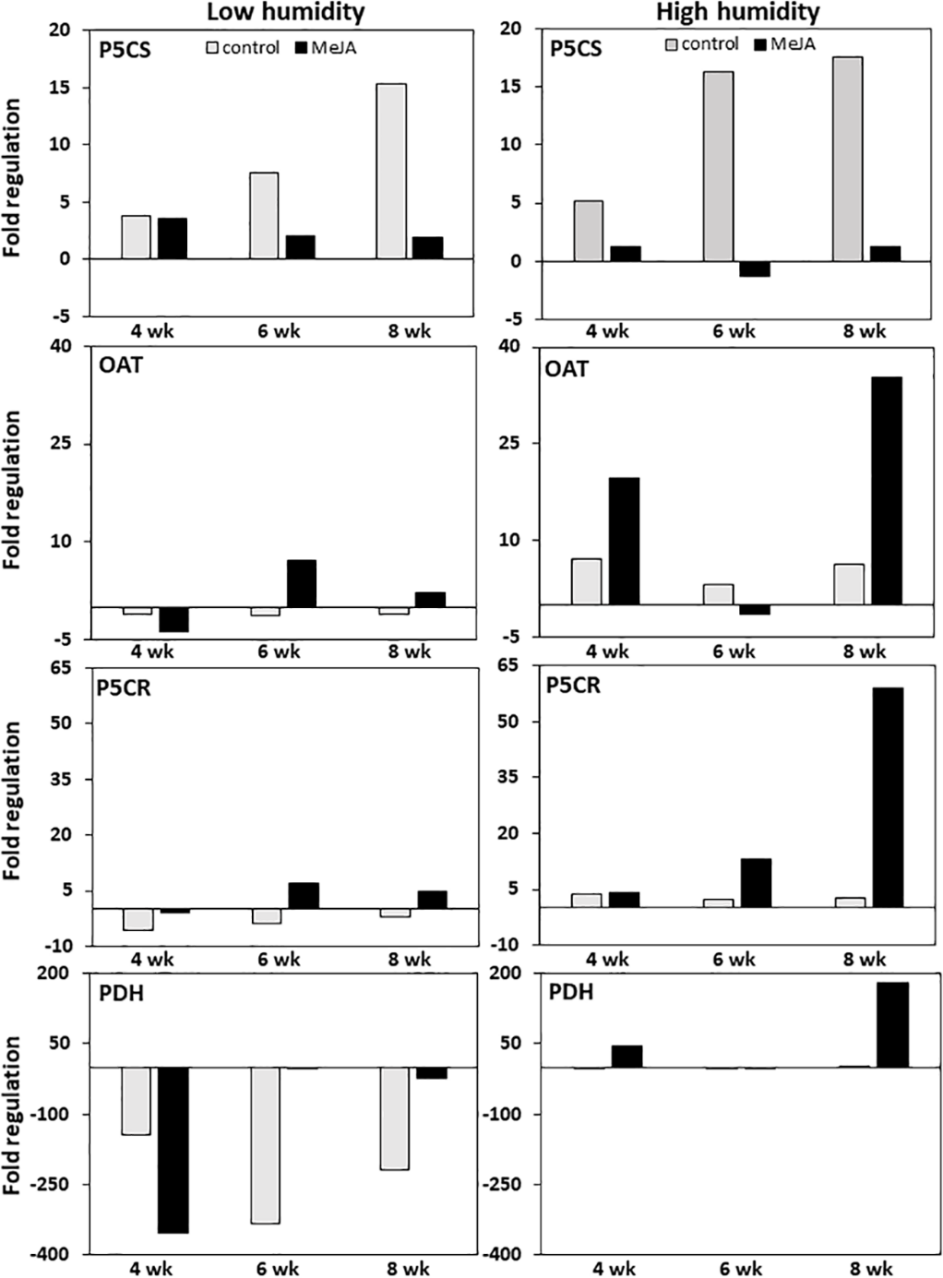
Changes in expression of genes involved in proline biosynthesis and catabolism in sugarbeet roots treated with methyl jasmonate (MeJA) or water (control) and stored at high and low relative humidity for up to 8 weeks. Expression of Δ^1^–pyrroline-5-carboxylate synthetase (P5CS), Δ^1^–pyrroline-5-carboxylate reductase (P5CR), ornithine *δ*-aminotransferase (OAT), and proline dehydrogenase (PDH) was determined relative to expression on the day of harvest. Roots were treated with 10 µM MeJA or water after harvest and stored under high humidity (98% relative humidity) or low humidity (91% relative humidity) conditions at 20 °C.

MeJA and storage duration affected the expression of genes involved in proline biosynthesis and catabolism, with MeJA effects greatest in roots stored at high relative humidity (Fig. 4). In untreated controls, expression of P5CS, the gene responsible for the first committed step in the conversion of glutamic acid to proline, was upregulated approximately 15-fold during eight weeks in storage regardless of the humidity at which roots were stored. In contrast, expression of OAT, which catalyzes the first committed step in the conversion of ornithine to proline, and P5CR, which catalyzes the conversion of P5CS and OAT reaction products to proline, were minimally affected by storage duration at either humidity level. Treatment of roots with MeJA suppressed the upregulation of P5CS during storage such that P5CS increased by less than four-fold in MeJA-treated roots at either humidity level. In contrast, MeJA increased expression of both OAT and P5CR by as much as seven-fold under low humidity storage conditions, and by 35 and 60-fold for the same respective genes under high humidity conditions (Fig. 4).

Expression of proline dehydrogenase, the enzyme which initiates proline degradation to glutamic acid, was strongly affected by both humidity conditions and MeJA treatment (Fig. 4). Low humidity storage was associated with a large decline in PDH expression, such that PDH transcripts in low-humidity control roots declined by as much as 340-fold after six weeks of storage but remained unchanged in high-humidity control roots. Additionally, MeJA altered the response of PDH expression to humidity conditions. Under low humidity, MeJA hastened downregulation of PDH, with PDH transcripts in MeJA-treated roots reduced by 350-fold after four weeks in storage versus the six weeks needed to obtain a similar downregulation in control roots. Following maximum repression, the decline in PDH transcripts was attenuated in both control and MeJA-treated roots. However, the alleviation of PDH repression occurred more rapidly and to a greater extent in MeJA-treated roots than in untreated controls. Under high humidity conditions, MeJA upregulated PDH expression relative to the control treatment, with expression increased by as much as 175-fold in MeJA-treated roots stored for eight weeks. Overall, gene expression related to proline degradation was affected to a much larger extent by MeJA treatment and storage conditions than was the expression of any genes that contribute to proline biosynthesis.

## Discussion

Storage of sugarbeet roots under low relative humidity conditions led to large reductions in root weight and significant elevations in respiration rate. The loss in root weight was primarily due to water loss which increases in direct proportion to the water vapor pressure gradient between the root surface and the surrounding air (Ben-Yehoshua & Rodov, 2002). Although respiration converts carbon-containing compounds to carbon dioxide, weight loss from this conversion is minimal (Kays & Paull, 2004). Root respiration rate increased with even low levels of dehydration, and after one week in storage, roots that had lost less than 5% of their weight respired at a rate that was 2.5-fold greater than well-hydrated roots stored at high humidity. Respiration rate further increased as dehydration became more severe, and after 6 to 8 weeks storage under low-humidity conditions, respiration rate increased 50%, as root weight loss increased from 22 to 26%. Similar to these results, an earlier study found root respiration rate increased in proportion to weight loss during storage (Lafta & Fugate, 2009).

Although relative humidity during storage affected weight loss and respiration rate, it had no apparent effect on wound healing of sugarbeet roots. Vapor conductance at the injured apex of the root declined at similar rates for control roots stored under low or high humidity conditions. By measuring the rate of water loss from a surface, vapor conductance quantifies the creation of a water-impermeable layer at the site of injury (Lulai, Suttle & Pederson, 2008). For all roots, regardless of MeJA or humidity treatment, the decline in vapor conductance was largely complete after two weeks, consistent with a rate of wound-healing observed previously for sugarbeet roots stored at 12 °C (Fugate et al., 2016). That wound-healing in sugarbeet root was unaffected by the relative humidity of storage contrasts with the results of *Wiggington (1974)* who reported lower wound-healing rates in potato tubers stored at low humidity. The results of the present study, however, compare favorably with *Adams & Griffith (1978)* who reported little variation in potato wound-healing when relative humidity during storage exceeded 70%.

Proline accumulated in dehydrated roots, and after eight weeks storage, roots stored under low humidity conditions contained 71% more proline than those stored at high relative humidity. Proline accumulation is a well-documented response to dehydration in plants and effectively delays and alleviates dehydration stress by altering cytoplasmic osmotic potential, stabilizing membranes, scavenging reactive oxygen species, and protecting enzymes from dehydration-induced unfolding (Ashraf & Foolad, 2007; Hayat et al., 2012). Similar to the accumulation of proline in dehydrated postharvest sugarbeet roots in this study, proline also accumulates in leaves of young sugarbeet plants in response to drought stress (Fugate et al., 2018).

Proline accumulation in plants may arise from changes in proline biosynthesis and/or proline degradation (Kishor et al., 2005; Chun, Paramasivan & Chandrasekaren, 2018). In dehydrated sugarbeet roots, the transcriptional changes for genes involved in proline biosynthesis and catabolism suggest that proline accumulation in roots stored under low humidity was likely due to a reduction in proline degradation. Consequently, expression of PDH, the gene responsible for proline degradation, declined 340-fold in control roots stored under low humidity conditions but was unchanged from levels found at harvest in high-humidity roots. While expression of P5CS, the gene that catalyzes the presumptive rate-limiting step in the biosynthesis of proline from glutamate (Wang et al., 2017), increased approximately 15-fold in low-humidity stored roots, this gene was unlikely responsible for low-humidity proline accumulation since P5CS was similarly upregulated in high-humidity roots. Like dehydrated postharvest sugarbeet roots, proline accumulated in concert with repression of PDH activity in drought-stressed seedlings of maize (Rayapati & Stewart, 1991).

MeJA treatment of freshly harvested roots reduced weight loss from dehydration, but its effect was small and significant only in roots stored under high humidity conditions that experienced minimal levels of dehydration. MeJA did not significantly reduce weight loss in severely dehydrated roots stored at low humidity. Previously, MeJA treatment was found to effectively reduce dehydration in young, water-stressed sugarbeet plants (Fugate et al., 2018). In these water-stressed plants, MeJA alleviated losses in leaf water content but, in contrast to the results of this study, did so only under severe water stress conditions. Loss of water from harvested sugarbeet roots occurs primarily by transpiration through the root periderm (Ben-Yehoshua & Rodov, 2002). MeJA, however, was unlikely to limit periderm transpiration since vapor conductance measurements on unwounded surfaces of MeJA-treated and control roots were similar throughout storage under both humidity conditions (Table S2). Similarly, leaf transpiration rates were unaltered by MeJA treatment in young plants subjected to water stress (Fugate et al., 2018).

Despite having little effect on weight loss during storage, MeJA reduced respiration rate and vapor conductance from wounded surfaces, but only in roots stressed by low-humidity storage and only at limited times during the eight-week storage period. MeJA treatment reduced respiration rate in severely dehydrated roots that had been stored for 6 to 8 weeks under low humidity conditions. MeJA treatment, however, had no effect on root respiration rate at milder levels of dehydration. MeJA, therefore, only lowered respiration rate in roots that had lost over 20% of their fresh weight to dehydration. MeJA also reduced vapor conductance from the wounded surface of roots stored at low humidity, but only after vapor conductance had dramatically declined. MeJA, therefore, hastened wound-healing during the final stages in the development of a water-impermeable layer. That MeJA altered respiration rate and wound healing exclusively under dehydrating conditions is perhaps unsurprising since MeJA is a signaling compound for drought stress (Yang et al., 2019). It is also consistent with MeJA’s demonstrated ability in other plant species to alleviate drought stress symptoms and promote transcription of genes involved in cell wall repair of injured tissues (Moore et al., 2003; Wasternack, 2007; Yu et al., 2019).

MeJA did not significantly alter proline accumulation in stored sugarbeet roots regardless of humidity conditions or storage duration. Jasmonates were similarly found to have no effect on proline accumulation in other plant species and organs (Chen, Chou & Kao, 1994; Bandurska, Stroiński & Kubiś, 2003; Mir et al., 2018). Nevertheless, proline concentrations in sugarbeet roots generally increased with time in storage, especially under low humidity conditions. Although the mechanism by which this accumulation occurred is unknown, the increase in P5CS expression with prolonged storage suggests that proline synthesis via the glutamate to proline pathway may be involved.

Leaf regrowth from the vegetative buds present on the crown of the sugarbeet taproot negatively impacts root sucrose content, processing quality, and storability since leaves develop at the expense of stored sucrose, impede ventilation in storage piles, and are associated with increased accumulation of invert sugars and respiration rate (Wyse & Dexter, 1971; Beaudry, Stewart & Hubbell, 2011). Previously, it was observed that elevations in storage temperature promoted postharvest sprouting of sugarbeet roots (Wyse & Dexter, 1971; Beaudry, Stewart & Hubbell, 2011). Results of the present study, however, indicate that sprouting also depends on the relative humidity of storage conditions since sprouting was evident in roots stored under high humidity but absent on roots stored under low humidity conditions. Although sprouting was slightly reduced in response to a 10 μM MeJA treatment, MeJA had no statistically significant effect on the incidence or the growth rate of new leaves. In contrast, MeJA treatment effectively inhibited the sprouting of new leaves in topped radishes (Wang, 1998). Although the cause for the different sprouting responses of sugarbeet and radish taproots to MeJA treatment are unknown, MeJA concentrations that inhibited sprouting in radish were 10-fold or more in excess of those used in the present study. Similar to this study, *Oberg & Kleinkopf (2000)* found no effect of methyl jasmonate on sprout growth in potato.

## Conclusion

Although application of MeJA alleviates abiotic stress in numerous plant species and reduces drought stress in young sugarbeet plants, (Fugate et al., 2018; Yu et al., 2019), MeJA did little to alleviate the stress caused by root dehydration from low humidity storage conditions or improve storage traits in roots under non-dehydrating storage conditions. During eight weeks of storage, MeJA had little to no effect on dehydration and weight loss in storage, proline accumulation, or sprouting of vegetative buds under high or low humidity conditions and had no effect on root respiration rate or wound healing under high humidity conditions. Although MeJA lessened respiratory increases associated with dehydration and improved wound healing in low-humidity stored roots, these benefits were small and temporary with root respiration rate reduced only in the most severely dehydrated roots and wound healing hastened only in the final stages in the creation of a water-resistant barrier over the wound site. Therefore, while postharvest MeJA treatment may be useful for reducing losses due to storage rots (Fugate et al., 2012), it is unlikely to meaningfully alleviate sugarbeet root storage losses caused by dehydration.

### Declaration

Mention of trade names or commercial products is solely for the purpose of providing specific information and does not imply recommendation or endorsement by the US Department of Agriculture. USDA is an equal opportunity provider and employer.

##  Supplemental Information

10.7717/peerj.11623/supp-1 Supplemental Information 1 Regression equations describing the loss in weight for sugarbeet roots treated with methyl jasmonate (MeJA) or water (control) during eight weeks of storage under low humidity or high humidity conditions High humidity and low humidity conditions were 98% and 91% relative humidity, respectively. Weight loss is expressed as the reduction in weight as a percentage of root weight at harvest. Abbreviations: WL, weight loss; t, time in storage, expressed in weeks. Regression equations between MeJA and controls are significantly different at high humidity but are not statistically different at low humidity

10.7717/peerj.11623/supp-2 Supplemental Information 2 Raw data Measurements of (A) root respiration rate, weight loss in storage, vapor conductance, and proline concentration, and (B) Ct values that were used to determine gene expression changes for genes involved in proline metabolism.

## Additional Information and Declarations

### Data Availability

The following information was supplied regarding data availability: Raw data are available in the Supplementary File.
