# Supplementary material for: Methyl jasmonate effects on sugarbeet root responses to postharvest dehydration
Source: PeerJ. 2021 Jun 17;9:e11623. doi: 10.7717/peerj.11623 (PMC8214845; doi:10.7717/peerj.11623)
Supplement: Supplemental Information 1 — High humidity and low humidity conditions were 98% and 91% relative humidity, respectively. Weight loss is expressed as the reduction in weight as a percentage of root weight at harvest. Abbreviations: WL, weight loss; t, time in storage, expressed in weeks. Regression equations between MeJA and controls are significantly different at high humidity but are not statistically different at low humidity [file peerj-09-11623-s001.docx]

**Supplementary File S1**

Regression equations describing the loss in weight for sugarbeet roots treated with methyl jasmonate (MeJA) or water (control) during eight weeks of storage under low humidity or high humidity conditions. High humidity and low humidity conditions were 98% and 91% relative humidity, respectively. Weight loss is expressed as the reduction in weight as a percentage of root weight at harvest. Abbreviations: WL, weight loss; t, time in storage, expressed in weeks. Regression equations between MeJA and controls are significantly different at high humidity but are not statistically different at low humidity.

| **Treatment** | **Storage conditions** | **Equation** | **R^2^** | **Significance** |
| --- | --- | --- | --- | --- |
| MeJA | low humidity | WL = -0.2242t^2^ + 4.8735t | 0.99 | ns |
| Control | low humidity | WL = 0.2236t^2^ + 5.0797t | 0.99 | ns |
| MeJA | high humidity | WL = 0.8323t | 0.99 | ** |
| Control | high humidity | WL = 1.0038t | 0.99 | ** |

ns,**: Nonsignificant and significant at 1% probability by t-test
